# The importance of ClinicalTrials.gov in informing trial design, conduct, and results

**DOI:** 10.1017/cts.2025.9

**Published:** 2025-02-25

**Authors:** Robert M. Califf, Tracy L. Cutler, Hilary D. Marston, Ann Meeker-O’Connell

**Affiliations:** 1 Department of Medicine, Duke University School of Medicine, Durham, NC, USA; 2 U.S. Food and Drug Administration, Silver Spring, MD, USA

*The ClinicalTrials.gov data bank affords insight into clinical research questions, variability in trial designs, and research outcomes and can also provide data to improve the efficiency of evidence generation. The U.S. Food and Drug Administration (FDA) is working to advance compliance and enforcement activities where it has authority while also calling for broader participation of institutions in compliance to achieve a more useful, comprehensive data bank.*

ClinicalTrials.gov, a public data bank maintained by the National Library of Medicine, represents a national effort to improve transparency in research involving human participants. Launched in 2000, ClinicalTrials.gov was intended to provide consumer-friendly information about available clinical trials, particularly those evaluating experimental drugs for patients with serious and/or life-threatening diseases or conditions [1]. The Food and Drug Administration Amendments Act of 2007 (FDAAA) broadened the scope of ClinicalTrials.gov to include other types of clinical trials, increase transparency regarding trial design, and enhance public dissemination of study outcomes [2].

At FDA, we are constantly seeking to improve the quality and efficiency of clinical trials and other forms of human research to improve our regulatory function and support more effective clinical practice. A complete record of clinical trials is a desirable attribute, one that enhances the value of ClinicalTrials.gov as a key resource for analysis of trials and improvement of the clinical trials enterprise. Here, we review the current and potential value of this asset and the importance of broader participation of institutions in compliance that goes beyond requirements established by FDA to achieve a more useful, comprehensive database. We are concerned that excessive focus on FDA compliance activities diverts from broader efforts needed from relevant institutions.

The ethical foundation for ClinicalTrials.gov has been described previously [3]. Well-designed clinical trials can create generalizable knowledge for societal benefit. However, it is difficult to argue that this goal is served when a study and/or its results are not disclosed. Transparency in clinical research, including disclosing trial outcomes, is an important part of the ethical obligations to research participants described in the Declaration of Helsinki. Transparency informs patients and clinicians about planned and ongoing research, assists in identifying potential trials for participation, and offsets negative reporting bias in which trials with outcomes that fail to meet their objectives are less likely to be published.

As elements such as study design and outcomes have been added to ClinicalTrials.gov, it can increasingly serve as a source of information to form a basis for analyses to inform future trial design and to help set priorities. For example, evaluating trials that fail to answer a relevant question or enroll an adequate sample size could help researchers design trials that are more likely to succeed in their aims [4]. In addition, analysis of ClinicalTrials.gov data (both ongoing and completed research) can identify knowledge gaps that need to be filled with new trials and avoid duplicative efforts, potentially enabling substantial improvements in the efficiency of the clinical research enterprise while also avoiding exposing research participants to risk when the answer to a research question is already known. Multiple committees, reports, and publications have highlighted inadequacies in protocol design and statistical analysis plans. Seizing opportunities to share and analyze the data bank elements [5] could enable progress in these areas. For example, fields for informed consent documents were recently added. Sponsors and trialists whose examples of clear, concise, participant-oriented informed consent forms are accessible through the data bank are creating a valuable resource for analysis so that the broader field can iterate to the most effective approaches for informed consent documents (Table 1).

**Table 1.** Examples of uses of ClinicalTrials.gov information*

• Bring forth data from negative trials	• Evaluate common issues leading to trial termination
• Review and analyze submitted study documentation	• Help set priorities for research for a particular indication or medical condition
• Inform future trial design	• Prevent duplicative research efforts
• Identify knowledge gaps	• Allow patients and clinicians to search for research studies

*This a summary if uses for ClinicalTrials.gov information and is not meant to serve as an exhaustive list.

For these reasons, there is widespread interest about whether ClinicalTrials.gov fulfills these goals. Consistent, timely, and accurate compliance with the ClinicalTrials.gov submission requirements is expected but debate exists about how well investigators and institutions satisfy these requirements. Industry sponsors are clearly improving compliance year after year [6]. Although academic and institutional sponsors are also improving, they continue to lag behind industry [6].

As of July 2024, ClinicalTrials.gov provides information for more than 500,000 clinical studies. However, most of these – including observational studies, behavioral intervention studies, phase 1 drug trials, medical device feasibility studies, and studies of FDA-regulated products other than drug products, biological products, and medical devices (e.g., tobacco) – are not subject to FDAAA’s ClinicalTrials.gov requirements. FDAAA authorizes FDA to enforce compliance with relevant provisions for a subset of interventional clinical trials that study FDA-regulated drug products, biological products, and medical devices. Specifically, section 801 of FDAAA, including its implementing regulations at 42 CFR Part 11 (effective 1/18/2017), specifies requirements for submitting registration and summary results information for studies meeting the definition of an applicable clinical trial (ACT) (Table 2).

**Table 2.** Criteria for a clinical study to be considered an applicable clinical trial*

Study must be interventional (a clinical trial)
Study must evaluate at least one FDA-regulated drug, biological, or device product
Study must be other than: 1) A phase 1 trial of a drug or biological product, OR 2) A small clinical trial evaluating device feasibility or testing a prototype device
Study must meet at least one of the following: 1) At least one study facility located within U.S./U.S. territory, OR 2) Conducted under an U.S. FDA investigational new drug application (IND) or investigational device exemption (IDE), OR 3) Involves a drug, biological, or device product manufactured in and exported from U.S./U.S. territory for study in another country.

*An Applicable Clinical Trial must be registered (42 CFR 11.22(b)) and summary results information submitted (42 CFR 11.42) to ClinicalTrials.gov.

ACTs must be registered in ClinicalTrials.gov by the trial’s responsible party (RP) (42 CFR 11.22(b)). The sponsor (the entity that takes responsibility for and initiates the study) is usually the RP, unless the sponsor designates a principal investigator as the RP (42 CFR 11.4(c)). RPs must also submit summary results information (42 CFR 11.42), which is generally due no later than 1 year after the ACT’s primary completion date. However, before the deadline for summary results information submission, RPs may submit a certification for delayed results information submission (which could delay submission of summary results information for up to 2 years after the primary completion date for a trial conducted to support approval of a new product or new indication for an approved product) (42 CFR 11.44(c)). RPs may also request a “good cause” extension (42 CFR 11.44(e)) or waiver (42 CFR 11.54)).

Despite substantial progress since FDAAA’s enactment and implementation, concerns persist regarding the extent, effectiveness, and visibility of the FDA’s compliance and enforcement activities [6]. FDA encourages RPs to voluntarily comply with their legal obligations regarding ClinicalTrials.gov. The agency’s Bioresearch Monitoring Program uses a risk-based approach to prioritize compliance and enforcement activities regarding ClinicalTrials.gov according to potential for public health impact or risk to research participant safety, while also balancing resource needs across all compliance programs monitoring FDA-regulated trials.

When considering FDA’s compliance and enforcement activities for ClinicalTrials.gov, it is important to remember that the majority of registered studies are not ACTs and therefore are not subject to ClinicalTrials.gov reporting requirements and fall outside of the FDA’s enforcement authority. In addition, FDA considers non-public information submitted to the agency as part of investigational or marketing applications (e.g., protocols; clinical study reports) and information entered by RPs into ClinicalTrials.gov but not yet posted when evaluating whether a study is an ACT, and if so, whether potential enforcement action is appropriate. Other extenuating factors, such as an entity/RP becoming defunct, can also affect the FDA’s decision to pursue enforcement action.

A recent report by the Clinical Trials Transformation Initiative featuring interviews with clinical trial personnel identified multiple compliance challenges potentially affecting quality and timeliness of ACT registration and summary results information submission, chief among which was confusion about requirements and submission methods [7].

Thus, several issues deserve consideration. Submission of required summary results information would be improved if institutions and companies developed systematic approaches to support employed or affiliated RPs in meeting their responsibilities to submit such information, including when the RP is no longer able/available to meet reporting obligations. Given the significant resources needed to conduct clinical research, institutions and companies arguably share an interest in ensuring that submission requirements are met and understanding that transparency confers benefits that extend beyond compliance with FDAAA requirements. The Clinical Trials Transformation Initiative report also noted that prompt communication to study teams and the presence of a centralized administrative unit with experience submitting data to ClinicalTrials.gov were key to successful compliance [7].

Similarly, FDA has found that multistep compliance actions encouraging voluntary compliance are effective [8]. FDA is required to gather evidence supporting that a trial is an ACT before enforcing compliance. A preliminary internal analysis of curated ClinicalTrials.gov data found that <15% of registered studies initiated between January 18, 2017, and January 18, 2023, appear to meet ACT criteria and legal requirements for registration and submission of summary results information. FDA sends a Preliminary Notice of Noncompliance when it identifies potential noncompliance with ClinicalTrials.gov submission requirements and, after further review of information submitted to ClinicalTrials.gov, FDA may send a Notice of Noncompliance if potential violations are not addressed. This sequential enforcement activity has been very effective for those trials receiving notices.

Now is also an opportunity for institutions and companies to support registration and summary results information submission for all trials, which would alleviate confusion regarding which trials require reporting as ACTs. The vast arena of noninterventional clinical research would benefit from public posting of results information (as noted in current International Council for Harmonisation Guidelines), particularly given the use of real-world data in noninterventional study designs to generate real-world evidence that informs crucial medical and public-policy decisions. Transparency is also essential for the many interventional trials that fall outside the ACT definition and therefore are not subject to FDA enforcement under FDAAA. For these trials, there is no legal mandate, but we feel it is the responsibility of the institution and/or funding organization to encourage registration in ClinicalTrials.gov and subsequent reporting of research outcomes and for relevant investigators to make this a part of their overall effort to conduct trials. There are ethical issues raised when human experiments are done without making outcomes publicly available. This includes instances in which studies, including the plethora of observational studies of interventions, generate evidence contrary to financial interests or biases of the investigators and therefore are never made available to the public. As a clinical research community, we hope that institutions and firms will not only encourage public posting of study information in the absence of legal obligations but also put systems in place to ensure that posting happens.

ClinicalTrials.gov potentially affords insight into clinical research questions, variability in trial designs, and research outcomes. If used appropriately, it can also provide data to lead to approaches to improve efficiency of evidence generation so that clinical care, prevention, public health, and public-policy decisions are more often based on high-quality evidence. FDA recognizes the importance of ClinicalTrials.gov and will continue to advance compliance and enforcement activities where it has authority. We encourage sponsors, trialists, institutional officials, and the public to take full advantage of information available from ClinicalTrials.gov and other resources and to consider the research enterprise that exists beyond the domain of ACTs.
